# Nutritional status and its associated factors among infants and young children aged 6–23 months in Addis Ababa, Central Ethiopia, 2021: a cross-sectional study

**DOI:** 10.1017/jns.2024.20

**Published:** 2024-09-23

**Authors:** Sisay Hailu, Dube Jara, Eyob Ketema Bogale

**Affiliations:** 1 Hiddassie Health Center, Addis Ababa, Ethiopia; 2 School of Public Health, College of Health Sciences, Department of Biostatics and Epidemiology, Debre Markos University, Debre Markos, Ethiopia; 3 School of Medicine and Health Sciences, Department of Health Promotion and Behavioral Sciences, Bahir Dar University, Bahir Dar PO. Box, 079, Ethiopia

**Keywords:** Cross-sectional study, Ethiopia, Nutritional status, Yeka sub-city, Young children

## Abstract

The aim of this study is to assess nutritional status and associated factors among infants and young children aged 6–23 months in Yeka sub-city, Ethiopia, 2021. An institution-based cross-sectional study was conducted in selected health centres found in the Yeka sub-city from May 2021 to July 2021. In total, 396 systematically selected infants and young children aged 6–23 months attended the selected health centres were included in the study. Data were collected by using a structured questionnaire and anthropometric measurements. A multinomial logistic regression model was used.

The overall magnitude of undernutrition and overnutrition among infants and young children were 24.7% and 5.5%, respectively. Dietary diversity score (DDS) ((adjusted odd ratio (AOR) = 5.65; 95% CI = 2.301, 10.87; P value = 0.003), minimum meal frequency (MMF) (AOR = 5.435; 95% CI = 2.097, 11.09; P value = 0.0052), and diarrhoea (AOR = 2.52; 95% CI = 1.007, 6.310; P value = 0.002) were statistically significantly associated factors for nutritional status among infants and young children. Malnutrition (undernutrition and overnutrition) is a public health problem among infants and young children in Yeka sub-city, Ethiopia. DDS, MMF, and diarrhoeal disease were associated with higher odds of undernutrition.

## Background

Child malnutrition is a major public health and development concern in most poor communities leading to high morbidity and mortality throughout the world.^(1)^ Inadequate nutrition during the first 2 years of life leads to childhood morbidity including inadequate brain development and mortality. Infants are at increased risk of malnutrition by 6 months when breast milk alone is no longer sufficient to meet their nutritional requirements.^(2)^ Globally, 149.2 million children under 5 years of age are stunted, 45.4 million are wasted, and 38.9 million are overweight.^(3)^ The prevalence of undernutrition in the rural parts of Ethiopia was 47.6% stunted, 29.2% underweight, and 13.4% wasted.^(4)^ The prevalence of undernutrition in the urban parts of Ethiopia was 33.8% stunted, 12.6% underweight, and 8.3% wasted.^(5)^

The nutritional status of children from the lower socio-economic class was poor as compared to their counterparts in the upper socio-economic class.^(1)^ Many infants in sub-Saharan Africa start getting cereal-based supplemental feeds well before the age of 6 months, and in rare cases, they do not get them until the second year.^(2)^

Infant and young child feeding (IYCF) practices are multidimensional and change rapidly in short intervals in the first year of life, socio-demographic, and socio-economic factors and feeding practices affect the nutritional status of children aged 6 months and older. The Infant and Child Feeding Index is a composite index that measures complete feeding practices for infants and young children.^(6)^ Inappropriate IYCF practices in the first 2 years of life are among the major causes of childhood malnutrition in developing countries, including Ethiopia.^(7)^ Early introduction of solid foods and transitional feeding affect the early origin of weight gain and obesity risk.^(8)^

Poor feeding patterns, low socio-economic position, rural residency, insufficient health service coverage, and diseases are some of the factors that have an impact on nutritional status. A significant indicator of newborns’ and early children’s health status is their nutritional status.^(9)^ Children’s obesity is one of the twenty-first century’s most critical public health problems in the world.^(10)^

In Ethiopia, malnutrition is one of the most serious health and welfare problems among infants and young children commonly high prevalence of chronic malnutrition and undernutrition.^(11)^ Undernutrition of infants and young children is affected by inappropriate IYCF practices.^(12)^ The nutritional status of infants and young children is affected by several factors including the socio-economic status of the family.^(13)^ The nutritional status of infants and young children is affected bilaterally, which results in overnutrition and undernutrition. Undernutrition has short-term and long-term impacts. The short-term impact of undernutrition is immunosuppression and increase risk of infection, which is caused by, inadequate food intake and repeated infection (diarrhoeal diseases and acute upper respiratory tract infection (AURTI)) and also, the long-term impact of undernutrition is growth and development retardation, decreased school performance, and decreased productivity at a later age.^(14)^ Infants and young children who initiate early mixed feedings with breast milk are more likely to become obese at a young age.^(8)^

Even though there are a number of studies conducted in different parts of the world, there is limited information on the magnitude of nutritional status and associated factors among infants and young children aged 6–23 months in Ethiopia. The objective of this study was to assess nutritional status and associated factors among infants and young children aged 6–23 months in order to address the aforementioned gap in the area.

## Methods and materials

### Study design and setting

An institutional-based cross-sectional study was conducted. This study was conducted in four selected health centres (Hiddassie Health Center, Yeka Abado Health Center, woreda 12 Health Center, and Woreda 13 Health Center) in the Yeka sub-city from May 2021 to July 2021. Yeka sub-city is one of the sub-cities located North East of Addis Ababa, Ethiopia.

### Sample size determination and sampling procedure

The sample size was calculated by using the formula for estimation of single population proportion, considering 43% prevalence,^(15)^ with a 95% CI and allowable error of 5%, and the final sample size was 396.

Study participants were selected using a systematic sampling technique among infants and young children, who were attending four selected health centres during the study period. Proportional allocation was used to maintain proportionality among the four selected health centres. A systematic sampling technique with a sampling interval of two was used to select infants and young children aged 6–23 months from each institution.

### Data collection procedure and measurement/instruments

Data were collected using an interviewer-administered structured questionnaire (supplementary file) and anthropometric measurements. Data were collected by trained clinical nurses. The interview was conducted in a separate room.

The questionnaire was adapted from previous literature and it was modified to the context of this study. The training was given to both supervisors and data collectors. The pre-test was conducted on 5% of the sample size at Hiddassie Health Center before the actual data collection period. A necessary correction was made based on the results of the pre-test data. The questionnaire was translated into the local language (Amharic) and back to English by fluent speakers of the two languages. Strict supervision was done by supervisors, and the overall quality of the data collection was also monitored by the principal investigator. The collected data were checked for completeness and consistency before starting, processing, and analysing data.

Dietary diversity score (DDS) and minimum meal frequency (MMF) were assessed using the 24-h dietary recall method. The MMF was fulfilled if the food was received two to three times per day at 6–9 months of age, three to four times per day at 9–11 months of age, and three to four times at 12–24 months of age, with additional nutritious snacks offered one to two times per day between meals in the last 24 h. The dietary diversity score was fulfilled if infants and young children consumed five or more food groups in the last 24hrs from the nine food groups.^(16)^

Weight and length were taken for each study participant. Length was measured using a length board and recorded to the nearest 0.1 cm. The bodyweight was measured using a weight scale when the participant wore light clothing, was barefoot, and recorded the weight to the nearest 0.1 kg. Measuring instruments were checked and calibrated before the procedure to make measurements more reliable. Finally, weight-for-length, weight-for-age, and length-for-age were also measured, and it was classified according to WHO classification to determine the nutritional status of the respondents.

This study was conducted according to the guidelines laid down in the Declaration of Helsinki and all procedures involving human subjects were approved by the Institutional Review Board of Addis Ababa Public Health Research and Emergency Management Directorate with reference number PHREM 1342/2021. Written informed consent was obtained from all participants.

### Operational definition

**Undernutrition**: When the body does not have adequate amount of one or more nutrients reflected in biochemical tests like haemoglobin level for anaemia, in anthropometric indicators such as stunting (low height-for-age) or wasting (low weight-for-height) and/or weight-for-age (underweight).^(17,18)^

**Wasting**: weight-for-length < −2 *Z* score of the median WHO child growth standards.^(17,18)^

**Stunting:** length-for-age < −2 *Z* score of the median WHO child growth standards.^(17,18)^

**Underweight:** weight-for-age < −2 *Z* score of the median WHO child growth standards.^(17,18)^

**Overweight:** weight-for-height > 2 standard deviations above the median.^(17,18)^

**Obese:** weight-for-height > 3 standard deviations above the median.^(17,18)^

**Complementary feeding:** refers to a process of introducing the infant to additional sources of nutrition other than breast milk, usually at the age of 6 months.^(19)^

**Dietary diversity score:** the standard guideline (FAO) for the individual determinant DDS should be focused on the amount and type of food consumed at the individual level.^(17,18)^

**Low family size:** family size means the number of persons counted as members of an individual’s household; when a family have four or fewer persons, we classify it as low family size.^(20)^

**Improved source of water:** improved drinking water sources are those which by nature of their design and construction have the potential to deliver safe water. This includes piped water, boreholes or tube wells, protected dug wells, protected springs, rainwater, and packaged or delivered water.^(21)^

**Improved latrines:** are those designed to hygienically separate excreta from human contact. These include wet sanitation technologies (flush and pour flush toilets connecting to sewers, septic tanks, or pit latrines) and dry sanitation technologies (ventilated improved pit latrines, pit latrines with slabs, or composting toilets).^(22)^

**Good solid waste management:** good solid waste management practices are considered a success in properly segregating solid waste and/or disposing of it in an authorized location.^(23)^

**Diarrhea:** having three or more loose/watery stools in a 24-h or more loose/watery stool than are normal for the individual as entirely reported by the mother/caretaker of the child.^(24)^

**Occupation:** is defined as the main work undertaken by the participant/husband. If a participant/husband has more than one job, we report their main job.^(25)^

### Ethics approval and consent to participate

Ethical approval was obtained from the Addis Ababa Public Health Research and Emergency Management Directorate with a reference number PHREM 1342/2021. Permission was obtained from concerned stakeholders. Written informed consent was obtained from parents (mothers/caregivers) of children who attended a selected health centre, in the Yeka sub-city during data collection. The study participants were assured of confidentiality by excluding their names during the period of data collection. The rights were given to study participants to refuse, stop, or withdraw from the interview at any time. Confidentiality was maintained throughout the study.

### Statistical analysis

The data were coded, entered using Epi-Data Version 3.1, and exported to SPSS version 25 for analysis, and anthropometric measurements were measured for WHO *Z* score classification using WHO Anthro plus software (version) v3.2.2. The descriptive summary was presented using frequencies, proportions, figures, and tables. A multinomial logistic regression model was used to analyse the association. Both the bi-variable and multinomial logistic regression analyses were performed to assess the association between dependent and independent variables. All covariates with a P value <0.25 during bi-variable analysis were considered for further multinomial logistic regression analysis to control possible confounders and identify true predictors of nutritional status. Finally, those variables that showed a P value < 0.05, with 95% CI and adjusted odds ratio were considered to declare the variables were significantly associated factors of the dependent variables.

## Results

### Socio-demographic characteristics of study participants

A total of 396 infants and young children who had attended the selected health centres were included for the study with a response rate of 96%. A total of 335 (88.2%) mothers belonged to the age group of 21–34 years, and 327 (86.1%) mothers delivered their child at the age of 21–34 years. A majority of 196 (51.6%) mothers had 1 child. Among them, mothers/caregivers who had 3 or fewer children, and 4 and above were 353 (92.9%) and 27 (7.1%), respectively. The age interval of delivery below 2 years was 8 (2.1%) and three and above was 176 (46.3%). A majority of 371 (97.6%) mothers were married and 321 (84.5%) had a low family size.

About half, 191 (50.3%) of the mothers were educated at the primary educational level, 53 (13.9%) of mothers were unable to read and write, and 136 (35.8%) of mothers were educated at the secondary and higher educational level. A majority of 318 (83.7%) mothers were unemployed and 293 (77.1%) of mothers were housewives by their occupation. About three-fourth, 297 (78.2%) of mothers had <1000-birr monthly income. A majority of 191 (50.3%) of the husbands were educated at the secondary educational level. A majority of 233 (61.3%) of the husbands were unemployed and 123 (32.4%) of the husbands were in daily labour. A majority of 203 (53.4%) of the husbands have 1000–5000-birr monthly income (Table 1).

**Table 1.** Socio-demographic characteristics of respondents and infants and children characteristics in Yeka sub-city, Ethiopia, 2021 (*n* = 380)

Variables	Category	Frequency	Percentage
Age of mother	15–20 years 21–34 years >34 years	8 335 37	2.1 88.2 9.7
Maternal age at the time of delivery	15–20 years 21–34 years >34 years	33 327 20	8.7 86.1 5.3
Marital status	Married Divorced	371 9	97.6 2.4
Number of children	Three and less than three Four and more	353 27	92.9 7.1
Birth spacing	No age interval Two and more Less than 2 years	196 176 8	51.6 46.3 2.1
Number of family size	Four and less Five and more	325 59	84.5 15.5
Educational level of mother	Unable to read and write Primary Secondary and higher educational level	55 189 136	14.5 49.7 35.8
Mother/caregiver occupation	Housewife Government and private employed worker Others	288 66 26	75.8 17.4 6.8
Mother’s monthly income (birr)	Had no income by own <1000 1000–5000 >5000	281 14 49 36	73.9 3.7 12.9 9.5
Husband’s educational level	Unable to read and write Primary Secondary and higher educational level	39 151 190	10.3 39.1 50
Husband’s occupation	Government and private employed workers Daily labour Others	147 123 110	38 32.4 29.6
Husband’s monthly income (birr)	<1000 1000–5000 >5000	26 203 151	6.8 53.4 39.7
**Infants and children characteristics**	**Age** 6–11 months 12–17 months 18–23 months	82 182 112	22.6 47.9 29.5
	**Sex** Male Female	194 186	51.1 48.9

### Infant and young child characteristics

Of the total 194 (51.1%) infants and young children, 48.9% were male. Approximately 47.9% of children belong to 12–17 months. The mean age of the children was 14.7 months (±SD 4.4) (Table 1).

### Breastfeeding practices

A majority of 347 (91.3%) mothers currently practiced breastfeeding. Among mothers who currently practiced breastfeeding, 81.1% practiced exclusive breastfeeding to their infants. A majority of 347 (91.3%) mothers breastfed after 6 months, 19 (5%) infants and young children were fed <6 months, and 14 (3.9%) never breastfed. A total of 132 (34.7%) infants and young children were fed with bottle-feeding. Among the total bottle-feeding, eighty-two (21.6%) infants and young children started bottle-feeding after 6 months and fifty (13.2%) of them started bottle-feeding before 6 months. On the majority of them, 308 (81.1%) started at the appropriate time of complementary feeding than those who did not start, 72 (18.9%) (Supplementary Table 1).

### Dietary diversity score and minimum meal frequency

A majority of 362 (95.3%) infants and young children were fed starchy food. Fish and organ meat was poorly consumed by infants and young children. Dark green leaf and vitamin A-rich fruits and vegetables were consumed by 213 (56.1%) and 299 (78.7%), respectively. A majority of 266 (70%) infants and young children received adequate dietary diversity (DD). A majority of 335 (88.2%) infants and young children meet adequate MMF (Supplementary Table 2).

### Maternity and child health care services

A majority of 278 (73.4%) mothers used contraceptives and 101 (26.6%) mothers did not use contraceptives. All 380 (100%) mothers had ante natal care (ANC) follow-up during pregnancy and all of them delivered at health institutions. A majority of 362 (95.7%) mothers had post natal care (PNC) follow-up and 18 (2.3%) of them did not have PNC follow-up. All 380 (100%) infants and young children were vaccinated, and among them, 231 (60.8%) were fully vaccinated and 149 (39.2%) were vaccinated appropriately depending on their age. Among the total, fifty (13.2%) of infants and young children developed AURTI and forty-seven (12.4%) of them developed diarrhoeal disease (Supplementary Table 3).

### Hygiene and sanitation

On the majority, 353 (92.9%) of the mothers/caregivers have an improved source of water and 337 (88.7%) used improved latrines. A majority of 359 (94.5%) mothers/caregivers used a good solid waste management system disease (Supplementary Table 4).

### Nutritional status among infants and young children at the age of 6–23 months of study participants

**Fig. 1.**
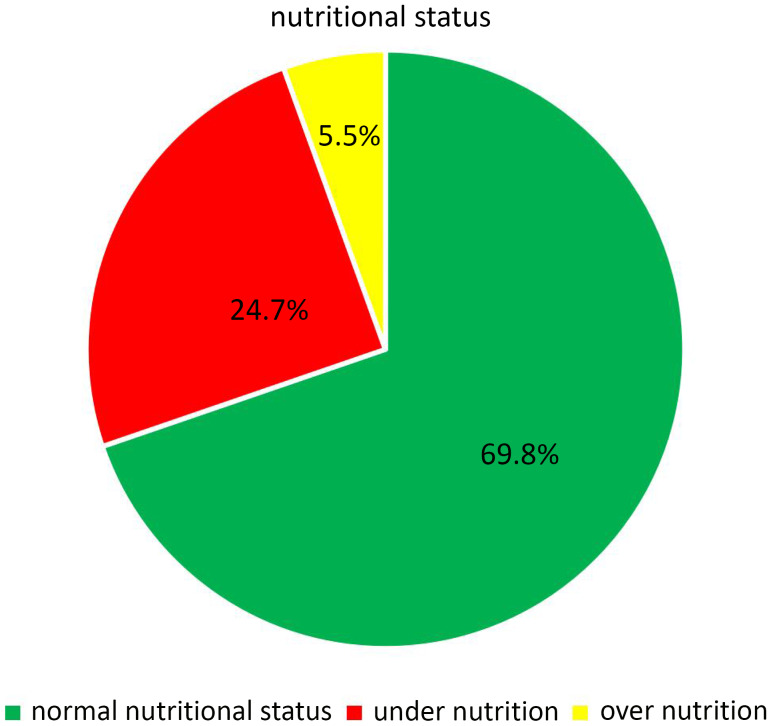
Nutritional distribution among infants and young children at the age of 6–23 months in Yeka sub-city, Ethiopia, 2021.

In general, ninety-four (24.7%) and twenty-one (5.5%) of infants and young children were undernourished and overnourished, respectively (Fig. 1).

### Anthropometric measurement result

**Fig. 2.**
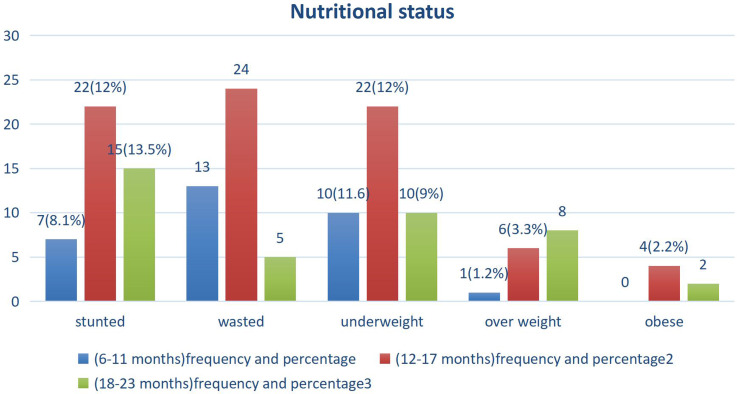
Nutritional status distribution among infants and young children at the age of 6–23 months in Yeka sub-city, Ethiopia, 2021.

The median and interquartile range of length, weight, and mid-upper-arm circumference (MUAC) of the study participants were 75.5 ± 8 cm, 9.5 ± 1.65 kg, and 13.6 ± 1.1 cm, respectively. Mean *Z* score values of infants and young children, WAZ, LAZ, and WLZ scores were −0.75, −1.25, and −0.26, respectively. The mean LAZ, WLZ, and WAZ scores were negative in all age groups (Fig. 2, Supplementary Table 5, and Supplementary Figs. 1–3).

### Factors associated with nutritional status among infants and young children

In binary logistic regression, maternal age at the time of delivery, occupation of the husband, starchy staples food, dark green vegetables, vitamin A-rich food, other fruits and vegetables, legumes, nuts, and seeds, milk and milk products, DDS, MMF, postnatal care visit, AURTI, diarrhoea, water source, latrine, and solid waste management were some of the determinant factors (with P value < 0.25) for undernutrition. Those variables having a P value of <0.25 in the binary analysis were taken to multinomial logistic regression analysis. In binary logistic regression, the age of the respondent, mother’s employment, husband’s employment, husband’s occupation, and DDS were some of the determinant factors (with P value < 0.25) for overnutrition. In multinomial logistic regression analysis, those variables with P value < 0.05 were considered significant predictors for undernutrition and overnutrition.

Multinomial logistic regression analysis results revealed that DDS (AOR = 5.7; 95% CI = 2.30, 10.87; P value = 0.003) was significantly associated with the nutritional status of infants and young children in this study. Infants and young children who did not obtain adequate DDS were more likely to develop undernutrition 5.7 times more than those who obtained adequate DDS (AOR = 5.7; 95% CI = 2.30, 10.87; P value = 0.003). As the DDS decreases by 1 unit, the odds of undernutrition risk increase by 5.7 times (Table 2).

**Table 2.** Factors associated with undernutrition among infants and young children using multinomial logistic regression in Yeka sub-city, Ethiopia, 2021 (*n* = 380)

Undernutrition							
Variables	Category	Frequency	COR	95% CI COR	AOR	95% CI AOR	P value
DDS	Inadequate Adequate	114 (30%) 266 (70%)	6.5 1	2.1, 6.6	5.65 1	2.3, 10.87	0.003
MMF	Inadequate Adequate	45 (11.8%) 335 (88.2%)	11.92 1	2.4, 12.1	5.44 1	2.1, 11.09	0.0052
Diarrhoea	Yes No	47 (12.4%) 333 (87.6%)	5.53 1	1.55, 7.02	2.52 1	1.0, 6.3	0.002
Overnutrition							
DDS	Inadequate Adequate	114 (30%) 266 (70%)	0.19 1	0.03, 1.45	0.14	0.02, 1.16	0.069
MMF	Inadequate Adequate	45 (11.8%) 335 (88.2%)	2.43 1	0.50, 6.76	4.08 1	0.77, 9.52	0.097
Diarrhea	Yes No	47 (12.4%) 333 (87.6%)	1.44 1	0.31, 6.69	1.48 1	0.31, 7.04	0.62

DDS, dietary diversity score; MMF, minimum meal frequency; COR, crude odds ratio; AOR, adjusted odds ratio; CI, confidence interval.

This study shows that MMF (AOR = 5.44; 95% CI = 2.10, 11.09; P value = 0.0052) was significantly associated with undernutrition. Infants and young children who did not meet adequate MMF are more likely to develop undernutrition 5.4 times than those who meet adequate MMF. Decreasing by one unit of MMF, there was increasing 5.4 times being odd of undernutritional status (Table 2).

The result of multinomial logistic regression analysis showed that diarrhoea within 2 weeks (AOR = 2.52; 95% CI = 1.01, 6.31; P value = 0.002) was significantly associated with the nutritional status of infants and young children. Children who developed diarrhoea within 2 weeks are more likely to develop undernutrition 2.5 times than those who did not develop diarrhoea. As the episode of diarrhoea increased by one unit being odd of undernourished increased by 2.5 times (Table 2).

## Discussion

In this study, the magnitude of undernutrition and overnutrition among infants and young children were 24.7% and 5.5%, respectively. DDS, MMF, and diarrhoea were significantly associated with nutritional status among infants and young children.

This study indicated that the magnitude of undernutrition and overnutrition were 24.7% and 5.5%, respectively. The finding was similar to the report of EDHS 2019, which was reported to be 21% and 4.5%, respectively.^(7)^ Among undernourished infants and young children 11.58%, 11.05%, and 11.05% were stunted, wasted, and underweight, and among overnourished, 3.95% and 1.2% were overweight and obese, respectively.

The magnitude of undernutrition among infants and young children in India was 65.2%, 43.3%, and 11.9% stunted, underweight, and wasted, respectively. When it was compared to this study, the magnitude of stunted and underweight was higher, due to poor DD and MMF practices, but in line with wasting prevalence.^(6)^ The magnitude of undernutrition among infants and young children in this study was consistent with a study conducted in rural China, which was 24.37%.^(26)^ The magnitude of underweight and stunted was lower in this study when compared to the study done in Harar, which were 21% and 19.3%, respectively.^(11)^ This might be due to the differences in these two studies where different determinant factors, such as breastfeeding practices, health status, and vaccination status of infants and children were associated factors that affect the nutritional status of infants and young children in Harar.

The prevalence of undernutrition in this study was lower than the prevalence of undernutrition in Tanzania.^(26,27)^ The cause of this difference was due to poor consumption of animal sources of food in Tanzania. The magnitude of stunted was high in Myanmar, which was 20%, due to poor DD and MMF.^(28)^ The magnitude of overnutrition in this study was 3.9% overweight and 1.6% obese, respectively, overweight was lower when it was compared to Kuwait, which was 6.5%, due to early initiation of complementary feeding that made the difference, but the prevalence of obese was similar to this study, which was 1.6%.^(29)^ The magnitude of stunted and underweight in this study was lower when it was compared to Ghana which were 20.5% and 21.1%, respectively.^(30)^ The factors that made the difference were poor DD and MMF which were 34.8% and 58.2%, respectively, in Ghana, but the magnitude of wasted was consistent, which was 11.5%.

The magnitude of undernutrition was low in this study when compared to research that was done in rural Ethiopia, which was 48.5%.^(31)^ The main cause of the difference was pre-lacteal feeding practices, high family and children size, and the high proportion of diarrhoea were significantly associated with the nutritional status of infants and young children.

The prevalence of overweight (WLZ > +2) and obesity (WLZ > +3) were 3.9% and 1.6% in this research, respectively, which were lower compared to the research done in South Africa, which were 11% and 5%, respectively.^(32)^ This might be due to anthropometric measurement difference which was used by MUAC for classification.

In this study, the magnitude of stunted was lower, which was 18.5% in Gamo Gofa, due to breastfeeding practice being highly related. Wasting was lower in Gamo Gofa, which was 3.9% when it was compared to this study. Underweight was consistent with this study which was 9.1%.^(33)^ The main difference was due to different determinant factors. Breastfeeding practices determined the magnitude of stunting in Gamo Gofa. In this study, the magnitude of stunted and underweight was lower when they were compared to the study which was done in Southern Ethiopia, which were 43.8% and 15.8%, respectively.^(34)^ The factor that made the difference was poor DDS compared to this study.

The magnitude of underweight, wasting, and stunting was lower than research which was done in Indonesia, which were 26%, 23%, and 28%, respectively.^(35)^ The factors that made the difference were poor DD and MMF. The magnitude of undernutrition in this study was lower when compared to the research done in Nigeria.^(36)^ The factors that made the difference were poor DDS and MMF compared to this study. The magnitude of undernutrition in this study was lower compared to that in Bule Hora, due to diarrhoea in the past 2 weeks and pre-lacteal feeding.^(4)^

DDS and MMF of this study were 70% and 88.2%, respectively, which were higher than the magnitude of minimum DD and MMF practices, which were 28.5% and 68.4% in Bale, Ethiopia, respectively.^(37)^ Similarly, DD and meal frequency were 17% and 72.2%, respectively, in Holeta town and 7% and 47%, respectively, in Bahir Dar.^(38,39)^ The DDS of this study was 70% which was nearly five times higher than the study that was done in Dejen, Ethiopia 13.6%.^(40)^ DD and meal frequency were 23.7% and 32.7%, respectively, in Pawie District, Benishangul Gumuz.^(41)^ They were lower than this study, due to the strong significance of the residence and PNC check-up. DD and meal frequency were 18% and 56%, respectively, in Ethiopia.^(42)^ They were lower than this study, mainly affected by poor timely initiated complementary feeding.

Minimum DD and meal frequency were 45% and 33%, respectively, in North Shoa, Ethiopia, which were lower than this study, due to postnatal care visits, child feeding practices, and getting media exposure.^(43)^ DD was 29.9% in North West Ethiopia,^(44)^ which was lower than this study; it was significantly associated with maternity services and child vaccination. The DDS of this study was 70% which was higher compared to 59.9% in Addis Ababa, Ethiopia.^(45)^ Increasing DD may be an approach to reduce the burden of stunting and chronic malnutrition among young children.^(46)^ DD and MMF were very low in Afar when compared to this study due to different predictors, maternal education, maternal occupation, sex of the child, and history of postnatal care visit, which were significantly associated factors.^(7)^

In this study, diarrhoea was significantly associated with the nutritional status of infants and young children. This finding is supported by studies conducted in Filtu town, Somali Region, Ethiopia,^(2)^ in Tigray,^(47)^ and rural parts of Ethiopia.^(4)^ Diarrhoea contributes to malnutrition through the reduction in food intake, decrease in absorption of nutrients, and increase in catabolism of nutrient reserves.

### Limitations of the study

A single 24-h recall dietary data might not reflect the usual intake of participants and recall bias was one of the limitations of this study. Moreover, the shortcomings of the cross-sectional study design may not enable the determination of causal relationships.

## Conclusion

High numbers of infants and young children suffered from malnutrition with low DDS, MMF, and diarrhoea. Large numbers of mothers/caregivers were housewives and did not get income on their own. As a result, infants and young children feeding practices were poorly practiced which was significantly associated with infants and young children nutritional status in the study area. The nutritional status of infants and young children should be assessed at a community level, so researchers should take the responsibility to determine the determinant factors by qualitative and quantitative study design.

## Supporting information

Hailu et al. supplementary material 1 Hailu et al. supplementary material

Hailu et al. supplementary material 2 Hailu et al. supplementary material

## Acronym and Abbreviations

**AA:** Addis Ababa; **DD:** Dietary Diversity; **ETB:** Ethiopian Birr; **FANTA:** Food and Nutrition Technical Assistance; **FAO:** Food and Agriculture Organization; **ICFI:** Infant and Child Feeding Index; **IDDS:** Individual Dietary Diversity Score; **IMNCI:** Integrated Management of Newborn and Childhood Illness; **EPI:** Ethiopia Program Immunization; **MAM:** Moderate Acute Malnutrition; **MUAC:** Mid-Upper-Arm Circumference; **NACS:** Nutrition Assessment; Counselling; and Support; **NNP:** National Nutritional Program; **OPD:** Outpatient Department; **OTP:** Outpatient therapeutic feeding program; **SAM:** Severe Acute Malnutrition; **WFP:** World Food Program; **WHO:** World Health Organization.

## Supplementary material

The supplementary material for this article can be found at https://doi.org/10.1017/jns.2024.20
