# Supplementary material for: Nutritional status and its associated factors among infants and young children aged 6–23 months in Addis Ababa, Central Ethiopia, 2021: a cross-sectional study
Source: J Nutr Sci. 2024 Sep 23;13:e43. doi: 10.1017/jns.2024.20 (PMC11440571; doi:10.1017/jns.2024.20)
Supplement: Hailu et al. supplementary material 1 — Hailu et al. supplementary material [file S204867902400020Xsup001.pdf]

Questionnaire (English Version)

**1. Sociodemographic and socioeconomic status**			
S/N	Questionnaires	Response category	
101	Age of mother/caregivers?	In year ………	
102	At what age you delivered your child?	In year …..	
103	What is your marital status?	1. Single<br>2. Marriage<br>3. Widowed<br>4. Divorced<br>5. Separated	
104	How many children do you have, if 1 pass to question number 106	1. Three and less<br>2. Four and more	
105	At what age interval delivered your children	1. Two and less years<br>2. Above two years	
106	How many family members live in the house	1. Five and less<br>2. Above five	
107	What is your educational level?	1. Unable read and write<br>2. Primary<br>3. Secondary and higher education	
108	Are you employed?	1. Yes<br>2. No	
109	What is your occupation?	1. House wife<br>2. Government<br>3. Private<br>4. Other specify	
110	What is your income you get per	In birr------	

	month?		
111	What is your husband level of education?	1. Unable to read and write<br>2. Primary<br>3. Secondary and higher education	
112	Does your husband is employed?	1. yes<br>2. No	
113	What is your husband's occupation?	1. Daily labor<br>2. Governmental<br>3. private<br>4. other (specify)	
114	What is your husband's income he gets per month?	In birr ……	
	**2.Breast feeding**		
201	Are you breastfeed currently?	1. Yes<br>2. No	
202	Had you breast feeding your child exclusively?	1. Yes<br>2. No	
203	For how long your child feed breast?	In months……	
204	Is your child feed bottle feeding? If no pass to Q 206	1. Yes<br>2. No	
205	If the answer is yes for number 204, when did you start bottle feeding?	In months ….	
206	When did you introduce your child of solid, semi-solid or soft food?	In months ……	
	Dietary diversity		
	**Food groups**	Example	Yes=1<br>No=0
207	STARCHY STAPLES	maize, rice, wheat,	

			sorghum, white tubers, roots		
218	DARK GREEN LEAFY VEGETABLES		dark green/leafy, kale, spinach, gommen		
209	VITAMIN A RICH		mangoes, papaya, pumpkin, carrots, squash, or sweet potatoes		
210	OTHER FRUITS AND VEGETABLES		tomato, onion, water melon, apple, lemon		
211	ORGAN MEAT		liver, kidney,		
212	FISH AND MEATS		fresh or dried fish or shellfish beef, pork, lamb, goat, wild game, chicken,		
213	EGGS		Chicken egg		
214	LEGUMES, NUTS AND SEEDS		beans, peas, lentils, nuts, seeds or foods made from these		
215	MILK AND MILK PRODUCTS		milk, cheese, yogurt or other milk products		
	**Meal frequency**				
216	How many times your child feed per a day?				
	Age	6-9	9-12		12-23	
	Breakfast	Snack	Launch	Snack	Dinner	Snack
**3. Health care characteristics**					
301	Have you ever used contraceptive?		1. Yes<br>2. No		
302	How many times did you visit antenatal clinic?		1. Missing<br>2. 1-3 times<br>3. Four and above		
303	Where did you deliver your child?		1. Home<br>2. Institutional		
304	Did you visit PNC clinic?		1. Yes		

		2. No	
305	Have your child been vaccinated?	1. Yes<br>2. No	
306	If yes the above question how many times your child vaccinated?	1. Appropriate age vaccinated<br>2. Not appropriate age vaccinated	
**3. Infectious status of infants and young children**			
401	Acute respiratory tract within two weeks	1. Yes<br>2. No	
402	Diarrheal diseases within two weeks	1. Yes<br>2. No	
**4. Water supply, environmental and personal hygiene conditions of the study participants**			
501	What type of water source have you used?	1. Piped<br>2. Public stand pipe<br>3. Borehole<br>4. Surface water<br>5. Tanker water<br>6. Unprotected spring<br>7. Other (specify)	
502	What type of toilet do you used?	1. flush<br>2. ventilated improved pit<br>3. open defecation<br>3. bucket latrine<br>4. hanging latrine	
503	What type of method have you used for solid waste management?	1. Community dumping<br>2. Burned<br>3. Others (specify)	
**5. Anthropometric measurement**			

601	Age of study participants in month	……in months	
602	Sex of study participants	1. Male<br>2. Female	
603	Birth date of child	DD/M/Y	
604	Height/length	………cm	
605	Weight	………cm	
606	MUAC	………cm	
