# Supplementary material for: Nutritional status and its associated factors among infants and young children aged 6–23 months in Addis Ababa, Central Ethiopia, 2021: a cross-sectional study
Source: J Nutr Sci. 2024 Sep 23;13:e43. doi: 10.1017/jns.2024.20 (PMC11440571; doi:10.1017/jns.2024.20)
Supplement: Hailu et al. supplementary material 2 — Hailu et al. supplementary material [file S204867902400020Xsup002.pdf]

Supplementary File

Table 1: Breast feeding practices characteristics among infants and young children in Yeka Sub-city, Ethiopia, 2021

Variables	Category	Frequency (n)	Percentage (%)
Currently breast feeding	No<br>Yes	33<br>347	8.7<br>91.3
Exclusively breast feeding	No<br>Yes	72<br>310	18.9<br>81.1
Breast feeding through out	Never breast feed<br>Breast feed more than six months<br>Feed less than six months	14<br>347<br>19	3.7<br>91.3<br>5
Bottle feeding	No<br>Yes	248<br>132	65.3<br>34.3
Duration of bottle feeding	Before 6 months<br>After 6 months	50<br>82	37.9<br>62.1
Complementary feeding	Inappropriate<br>Appropriate	72<br>308	18.9<br>81.1

Table 2: Dietary diversity and minimum meal frequency characteristics among infants and young children in Yeka Sub-city, Ethiopia, 2021

Dietary diversity	Category	Frequency (n)	Percentage (%)
Starchy staple	No<br>Yes	18<br>362	4.7<br>95.3
Dark green leafy vegetables	No<br>Yes	213<br>167	56.1<br>43.9
Vitamin A source foods	No<br>Yes	87<br>293	22.9<br>71.1
Other fruit and vegetables	No<br>Yes	81<br>299	21.3<br>78.7
Organ meat	No<br>Yes	336<br>44	88.4<br>11.6
Fish	No<br>Yes	333<br>47	87.6<br>12.4
Egg	No<br>Yes	271<br>109	71.3<br>28.7
Legumes, nut and seeds	No<br>Yes	70<br>310	18.4<br>81.6

Milk and milk products	No<br>Yes	123<br>257	32.4<br>67.6
Dietary diversity score	Inadequate<br>Adequate	114<br>266	30<br>70
Minimum meal frequency	Inadequate<br>Adequate	45<br>335	11.8<br>88.2

Table 3: maternal and child health care services characteristics among infants and young children in Yeka Sub-city, Ethiopia, 2021

Maternity health care services	Category	Frequency (n)	Percentage (%)
Contraceptive	No<br>Yes	101<br>279	26.6<br>73.4
ANC	No<br>Yes	 380	 100
Site of delivery	Home<br>Institution	 380	 100
PNC	No<br>Yes	18<br>362	4.3<br>95.7
Vaccination	No<br>Yes	 380	 100
For how long vaccinated	Fully<br>Age appropriate	231<br>149	60.8<br>39.2
AURTI	Yes<br>No	50<br>330	13.2<br>86.8
Diarrhea	Yes<br>No	47<br>333	12.4<br>87.6

ANC: Ante Natal Care, PNC: Post Natal Care, AURTI: Acute Upper Respiratory Tract Infection

Table 4: Hygiene and Sanitation among characteristics infants and young children in Yeka Sub-city, Ethiopia, 2021

Hygiene and sanitation characteristics	Frequency (n)	Percentage (%)
Water source		
Unimproved<br>Improved	27<br>353	7.1<br>92.9
Latrine		
Unimproved<br>Improved	42<br>338	11.1<br>88.9
Solid waste management		
Good<br>Poor	21<br>359	5.5<br>94.5

Table 5: Distribution of nutritional status based on Z-Score according to their age, among infants and young children in Yeka Sub-city, Ethiopia, 2021

	6-11 months	12-17 months	18-23 months	Total
**Stunting**				
Normal (≥-2SD)	79(91.9%)	160(88%)	97(86.5)	336(88.42%)
Stunted (<-2SD)	7(8.1%)	22(12%)	15(13.5%)	44(11.58%)
**Wasting**				
Normal(≥-2SD)	72(83.7%)	148(81.3%)	97(86.6%)	317(83.43%)
Wasted (<-2SD)	13(15.1%)	24(13.2%)	5(4.5%)	42(11.05%)
Overweight(≥2SD) and(<3SD)	1(1.2%)	6(3.3%)	8(7.2%)	15(3.9%)
Obese (≥3SD)	0	4(2.2%)	2(1.8%)	6(1.6%)
**Under weight**				
Normal(≥-2SD)	76(88.4%)	161(88%)	101(91%)	338(88.95%)
Underweight (<-2SD)	10(11.6%)	22(12%)	10(9%)	42(11.05%)

SD: Standard Deviation

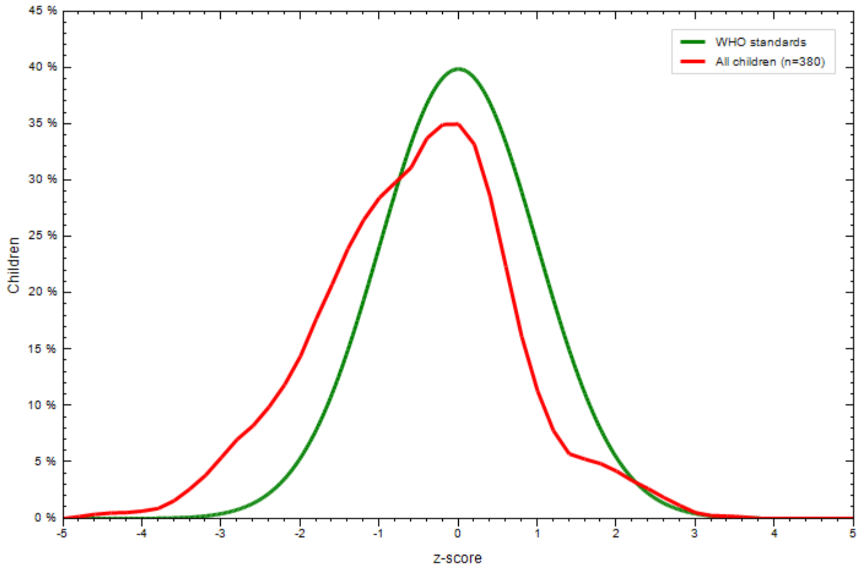

Figure 1. Distribution of under-weight (weight for age z score) among infants and young children the age of 6-23 months in Yeka Sub-city, Ethiopia, 2021

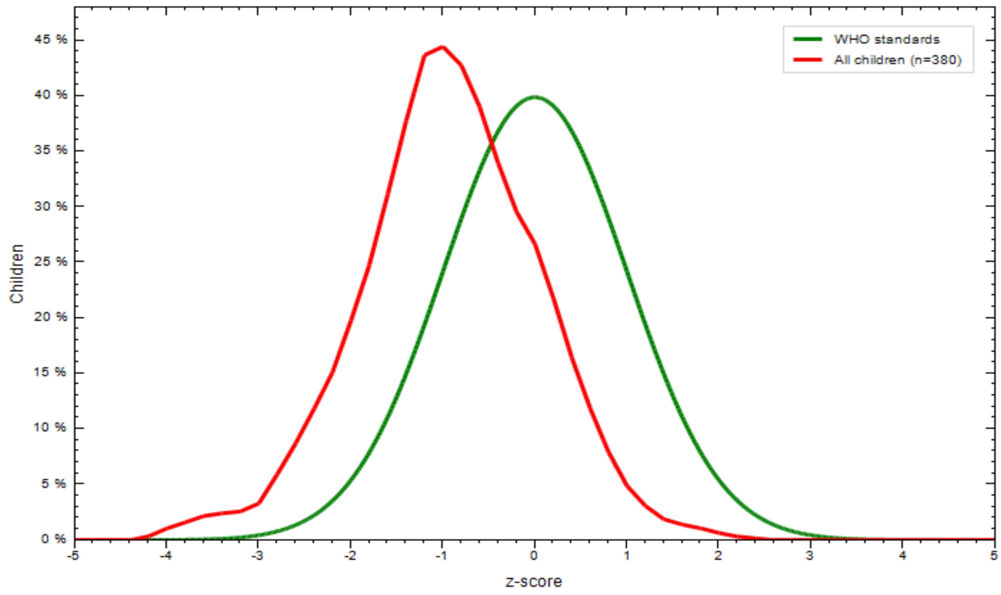

Figure 2. Distribution of stunting (length for age z score) among infants and young children the age of 6-23 months in Yeka Sub-city, Ethiopia, 2021

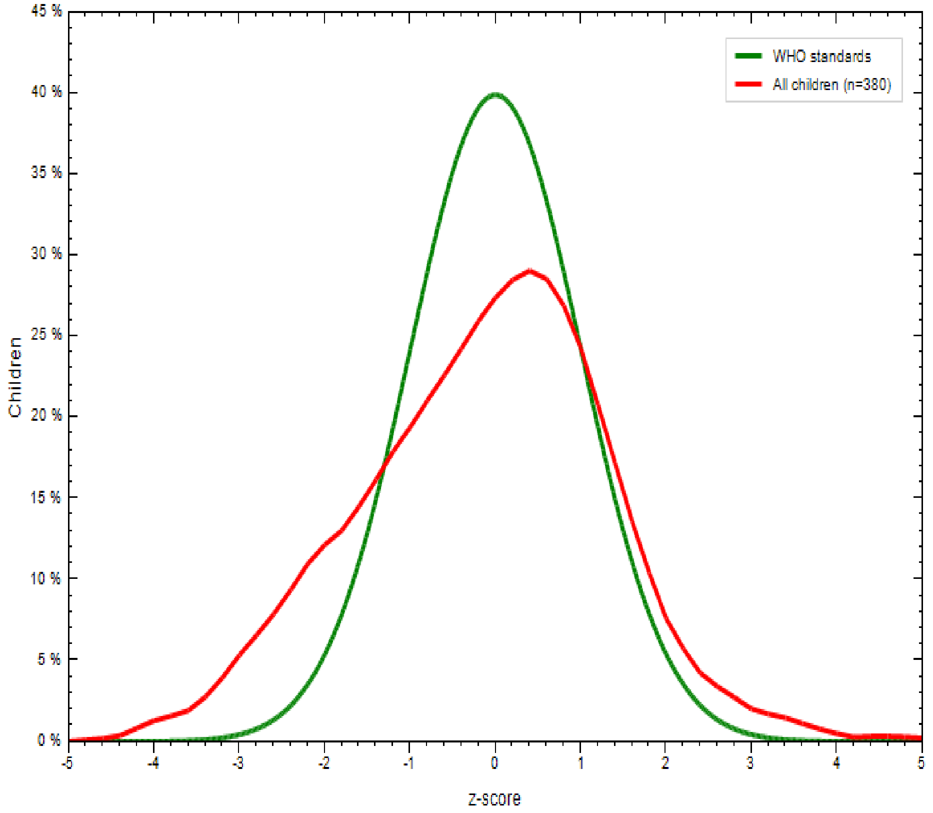

Figure 3. Distribution of wasting (weight for length Z-score) among infants and young children in Yeka Sub-city Ethiopia, 2021
